# Influencing factors of live birth in vitrified oocyte donation cycles: A retrospective cohort study

**DOI:** 10.1016/j.eurox.2026.100473

**Published:** 2026-06-27

**Authors:** Xiaojuan Wang, Yangfeng Yu, Qiuyun Yan, Juan Song, Ge Lin, Shuoping Zhang, Fei Gong

**Affiliations:** a Clinical Research Center for Reproduction and Genetics in Hunan Province, Reproductive and Genetic Hospital of CITIC-XIANGYA, Changsha, Hunan 410008, China; b NHC Key Laboratory of Human Stem Cell and Reproductive Engineering, Xiangya School of Basic Medical Sciences, Central South University, Changsha, Hunan 410008, China

**Keywords:** Donor oocyte, Frozen oocyte, Live birth rate, Influencing factor

## Abstract

**Objective:**

To evaluate the live birth rate (LBR) of assisted reproductive technology (ART) treatment using vitrified donated oocytes in China and to investigate the influencing factors on live birth outcomes.

**Methods:**

This retrospective cohort included 377 infertility women who underwent ART with vitrified donated oocytes at a large reproductive center in China between January, 2015 and June, 2024. Among them, 369 women underwent embryo transfer. The primary outcome was LBR after the first transfer. The multivariable Logistic regression model were employed to estimate the influencing factors of live birth outcomes.

**Results:**

The LBR of the first transfer was 51.5% (190/369). Advanced recipient age (adjusted odds ratio [aOR]: 0.92, 95% CI: 0.85–0.98, *P* = 0.015) and a history of previous poor oocyte/embryo quality (OR: 0.38; 95% CI: 0.18–0.75; *P* = 0.007) were associated with a reduced LBR. The cumulative live birth rate (CLBR) per initiated oocyte donation cycle was 58.5% (216/369). Advanced recipient age and a history of previous poor oocyte/embryo quality were associated with a reduced CLBR, and an increased number of day-3 usable embryos significantly improved the CLBR (OR: 1.47; 95% CI: 1.26–1.73; *P* = 0.000). Prolonged oocyte cryopreservation duration exerts no adverse impact on laboratory or clinical outcomes. Increased thawed oocytes hardly boost LBRs but raise surplus embryos.

**Conclusions:**

Advanced recipient age and a history of poor oocyte/embryo quality are significant factors reducing LBRs in ART treatment with vitrified donated oocytes. Long-term oocyte cryopreservation is safe, and rational batch oocyte allocation is recommended.

## Highlights

• This retrospective cohort analyzes live birth factors of vitrified donated oocyte in China to guide local clinical practice.
• Advanced recipient age mildly reduces live birth rate.
• Recipients with poor oocyte/embryo quality history show lower live birth rates.
• Prolonged oocyte cryopreservation duration exerts no adverse impact on laboratory or clinical outcomes.
• Full one-time oocyte allocation to a single recipient tends to create surplus embryos, so staged oocyte allocation is advised.

## Introduction

Approximately 15% of women of reproductive age experience infertility, which has become a significant global public health issue [1]. For women who may not be able to conceive due to diminished ovarian reserve, carrying significant genetic defects, or repeated poor embryo quality, oocyte donation-assisted reproductive technology (ART) offers a valuable opportunity to achieve pregnancy and build a family [2].

Regulations and clinical practice of oocyte donation vary widely across different countries and regions. In China [3], oocyte donors are limited to women aged 20–35 years who are undergoing IVF or ICSI treatment. Surplus oocytes can be donated only when the number of oocytes reserved for personal use reaches 15, and strict anonymity between donors and recipients is required by law. These regulations effectively prevent commercialization of oocyte donation and protect the rights and safety of both donors and recipients. However, they also lead to an extreme shortage of donated oocytes. According to the Chinese Society of Reproductive Medicine, oocyte donation cycles account for only 0.25% of all ART cycles in China, while the global average is approximately 9% [4]. Previous surveys have shown that approximately 85–95% of women who need donated oocytes for ART treatment are unable to obtain them in China [3]. Even in Europe, where donor sources are more diverse, a notable shortage of donated oocytes still exists [5]. Therefore, optimizing oocyte donation strategies and improving the success rate of ART using donated oocytes have become critical to improving oocyte utilization efficiency and helping more infertile families achieve their childbearing goals.

Since the widespread application of vitrification in oocyte cryopreservation, vitrified oocyte donation has greatly improved the flexibility and efficiency of oocyte donation programs. Previous studies have compared live birth outcomes between fresh and frozen oocyte donation cycles [6], and evaluated the effects of different cryopreservation techniques (slow-freezing vs. vitrification) [7], cryopreservation duration [8], [9], and other technical factors on laboratory and clinical outcomes. Most studies have confirmed that vitrification is a safe and effective method for oocyte cryopreservation. Other studies have explored the impacts of donor and recipient age [10], [11] and body mass index (BMI) [12], [13] on live birth rates. However, most of these studies only focused on individual factors and adjusted for a limited number of confounding variables, making it difficult to conduct a comprehensive assessment of factors influencing live birth. Fitzgerald et al. [14] developed a prediction model for live birth in oocyte donation cycles based on Australian population data. Nevertheless, oocyte donation regulations in Australia differ substantially from those in China, including donor age limits, donor sources, oocyte retention requirements, and directed donation policies. These differences result in distinct donor and recipient characteristics, limiting the generalizability of those findings to Chinese clinical practice.

This study aimed to evaluate the clinical outcomes of patients undergoing ART with vitrified donated oocytes under Chinese regulatory and clinical settings. We analyzed multiple factors associated with live birth, including donated oocyte characteristics, recipient conditions, and male partner factors. The purpose was to provide evidence for optimizing oocyte donation strategies and helping more infertile families achieve successful childbirth.

## Materials and methods

### Study design

A retrospective cohort study was conducted at the Reproductive and Genetic Hospital of CITIC-Xiangya. All patients who underwent ART using vitrified donated oocytes between January 1, 2015 and June 30, 2024 were enrolled. The follow-up period ended on June 30, 2025, and was terminated early if a live birth occurred or no embryos remained for transfer. Patients who underwent preimplantation genetic testing (PGT) were excluded from the analysis.

## Donor and recipient eligibility

In accordance with Chinese regulations and hospital standards, oocyte donors met all of the following criteria: (i) undergoing their first IVF cycle with more than 20 oocytes retrieved; (ii) aged 20–35 years; (iii) normal karyotype; (iv) no more than two spontaneous miscarriages; (v) negative results for infectious disease screening; (vi) no family history of hereditary or chromosomal diseases.

Recipients were eligible if they met at least one of the following indications: (i) ovarian failure; (ii) carrying significant genetic defects; (iii) history of poor oocyte or embryo quality.

Donors and recipients remained mutually anonymous. Each recipient receives oocytes solely from one single oocyte donor. All donated oocytes from the same donor are thawed at one time, and oocytes from one donor are only allocated to a single recipient.

## Laboratory procedures

All donated cumulus-oocyte complexes were cultured for 2–3 h before denudation and vitrification. Only metaphase II (MII) oocytes were vitrified using the Kitazato vitrification kit and Cryotop open straw, following the protocol described by Wang et al. [15]. One to two oocytes were vitrified in each straw. After thawing, oocytes were cultured for 2–3 h before intracytoplasmic sperm injection (ICSI). Oocyte survival was evaluated based on cytoplasmic morphology and membrane integrity. Normal fertilization was confirmed by the presence of two pronuclei (2PN) 16–18 h after insemination. Embryos were cultured in G1.5 medium (Vitrolife) in a tri-gas incubator (ASTEC APM−50D, Japan). On day 3, embryos were evaluated for cleavage and graded according to Puissant’s criterion. Day−3 usable embryos were defined as those with four or more cells and a fragmentation rate lower than 50%. High-quality embryos were defined as those with ≥ 6 cells and a fragmentation rate < 20%. If the patient had high-quality embryos, day 3 transfer was recommended, and the remaining embryos were cryopreserved. If the patient did not have high-quality embryos, it was recommended that the embryos underwent blastocyst culture followed by blastocyst transfer. Blastocysts were graded using the Gardner and Schoolcraft system on days 5–7 [16]. A maximum of two high-grade embryos were transferred into the recipient’s uterus, and surplus usable embryos were vitrified for subsequent transfers.

## Endometrial preparation for recipients

A conventional artificial cycle was typically used for endometrial preparation; however, if the patient had conditions such as adenomyosis or endometritis, a down-regulation artificial cycle was used. During the artificial cycle, oral estradiol was given in fixed doses or in a stepwise increasing manner for about 10 days. Progesterone was supplemented to facilitate endometrial transformation when endometrial thickness ≥ 8.5 mm and serum progesterone < 1 ng/mL. Embryo transfer was arranged on day 4 of luteal support for cleavage embryos and day 6 for blastocysts.

## Outcome definitions

The primary outcome was live birth of the first embryo transfer, defined as the delivery of a viable infant after the first embryo transfer. The secondary outcome was cumulative live birth, defined as at least one live birth per initiated oocyte donation cycle, which included all cycles utilizing fresh and/or frozen embryo transfer until a live birth occurred.

## Statistical analysis

Data were presented as mean ± standard deviation (SD) or frequency (%). Normality of continuous variables was tested using the Shapiro-Wilk test. Differences between groups were analyzed using two-sample *t*-test, Kruskal-Wallis test, or Pearson chi-square test, as appropriate. Multivariate logistic regression was used to identify independent factors associated with live birth after the first embryo transfer and cumulative live birth. Line charts were used to illustrate linear trends of live birth rate (LBR) with recipient age and oocyte cryopreservation duration. Linear trends were tested using linear regression models. The nonlinear relationship between cryopreservation duration and live birth was analyzed using restricted cubic spline (RCS) models based on logistic regression.

## Results

### Baseline characteristics

Between January 1, 2015 and June 30, 2024, a total of 380 infertile patients underwent thawing of vitrified donated oocytes. Three patients who underwent PGT were excluded, and 377 women were included in the final analysis. Among them, eight patients had no usable embryos, and 369 patients underwent the first embryo transfer, resulting in 190 live births and 179 non-live-birth cases. The live birth rate of the first embryo transfer cycle was 51.5% (190/369). The baseline and treatment characteristics of the live-birth and no-live-birth groups were presented in Table 1.

**Table 1:** Demographic and clinical characteristics of recipients according to live birth outcome.

	Overall (n = 377)	No-live birth (n = 187)	Live birth (n = 190)	*P*
Recipient age, years, mean ± SD	38.15 ± 6.45	38.88 ± 6.52	37.43 ± 6.31	**0.028**
Recipient BMI, kg/m^2^, mean ±SD	21.34 ± 1.78	21.36 ± 1.91	21.33 ± 1.65	0.867
Infertility years, years, mean ±SD	8.00 ± 4.93	7.90 ± 5.02	8.09 ± 4.85	0.707
Infertility type, n(%)				0.471
Primary	191(50.8)	91(48.7)	100(52.9)	
Secondary	185(49.2)	96(51.3)	89(47.1)	
Endometriosis, n(%)				1.000
No	328(87.0)	163(87.2)	165(86.8)	
Yes	49(13.0)	24(12.8)	25(13.2)	
Male age, years, mean (SD)	40.16 ± 7.03	40.66 ± 7.03	39.67 ± 7.02	0.172
Severe oligoasthenospermia, n(%)				0.343
Yes	34(9.0)	20(10.7)	14(7.4)	
No	343(91.0)	167(89.3)	176(92.6)	
Indication for donor oocyte use, n(%)				**0.040**
Poor ovarian reserve	268(71.1)	123(65.8)	145(76.3)	
Poor oocyte/embryo quality	46(12.2)	30(16.0)	16(8.4)	
Genetic factor	63(16.7)	34(18.2)	29(15.3)	
Duration of oocyte cryopreservation, months, mean ±SD	29.47 ± 15.24	29.38 ± 15.83	29.57 ± 14.68	0.905
Number of thawed oocytes, mean ±SD	5.68 ± 1.17	5.70 ± 1.16	5.66 ± 1.18	0.757
Number of day3 usable embryos, mean ±SD	3.62 ± 1.53	3.40 ± 1.68	3.83 ± 1.33	**0.006**
Number of transferred embryos, n(%)				**0.004**
0	8(2.1)	8(4.3)	0(0.0)	
1	25(6.6)	16(8.6)	9(4.7)	
2	344(91.3)	163(87.2)	181(95.3)	
Type of transferred embryos, n(%)				0.298
Cleavage stage	349(94.3)	167(92.8)	182(95.8)	
Blastocyst stage	21(5.7)	13(7.2)	8(4.2)	
Endometrial preparation protocol, n(%)				0.206
Natural cycle	63(16.7)	32(17.1)	31(16.3)	
Artificial cycle	156(41.4)	71(38.0)	85(44.7)	
Down-regulation artificial cycle	92(24.4)	54(28.9)	38(20.0)	
Others	66(17.5)	30(16.0)	36(19.0)	
Endometrial thickness before embryo transfer, mm, mean ±SD	11.36 ± 1.33	11.28 ± 1.40	11.45 ± 1.25	0.228

## Influencing factors of live birth of the first embryo transfer

Multivariate logistic regression analysis of the first embryo transfer cycles (as shown in Table 2) showed that advanced recipient age was associated with a lower LBR (Odds Ratio [OR]: 0.92; 95% Confidence Interval [CI]: 0.85–0.98; *P* = 0.015). Recipients with a history of poor oocyte/embryo quality had significantly lower LBR compared with those with diminished ovarian reserve (OR: 0.38; 95%CI: 0.18–0.75; *P* = 0.007). Factors including infertility duration, infertility type, endometriosis, recipient BMI, male age, number of thawed oocytes, number of day−3 usable embryos, oocyte cryopreservation duration, severe oligoasthenozoospermia, embryo transfer type, endometrial preparation protocol, and endometrial thickness before transfer showed no significant effects on LBR.

**Table 2:** Logistic regression analysis of live birth in the first embryo transfer cycle.

	Estimate	OR (95%CI)	*P*
Recipient age	−0.088	0.92(0.85,0.98)	**0.015**
Recipient BMI	0.026	1.03(0.90,1.17)	0.703
Infertility years	0.020	1.02(0.97,1.07)	0.421
Infertility type	0.363	1.44(0.84,2.50)	0.193
Endometriosis	0.447	1.56(0.77,3.24)	0.221
Male age	0.028	1.03(0.97,1.09)	0.378
Severe oligoasthenospermia	−0.285	0.75(0.34,1.62)	0.468
Indication for donor oocyte use			
Poor ovarian reserve	Ref		
Poor oocyte/embryo quality	−0.974	0.38(0.18,0.75)	**0.007**
Genetic factor	−0.598	0.55(0.28,1.07)	0.079
Duration of oocyte cryopreservation	0.008	1.01(0.99,1.02)	0.319
Number of thawed oocytes	−0.119	0.89(0.72,1.09)	0.255
Number of day−3 usable embryos	0.159	1.17(1.00,1.38)	0.053
Number of transferred embryos	0.596	1.81(0.56,6.22)	0.322
Type of transferred embryos	−0.113	0.89(0.25,3.27)	0.862
Endometrial preparation protocol			
Natural cycle	Ref		
Artificial cycle	0.015	1.02(0.52,1.97)	0.965
Down-regulation artificial cycle	−0.391	0.68(0.32,1.43)	0.307
Others	0.111	1.12(0.52,2.39)	0.773
Endometrial thickness before embryo transfer	0.097	1.10(0.93,1.31)	0.259

Poor oocyte/embryo quality was further classified into four categories: oocyte maturation defect, fertilization defect, poor-quality cleavage-stage embryo and blastocyst formation defect. As shown in Table 3, recipients with a history of poor-quality cleavage-stage embryo or blastocyst formation defect had significantly lower LBRs compared to other recipients.

**Table 3:** Live birth rates in the first embryo transfer cycle by indication for using donated oocyte.

Indication for using donated oocyte	Recipients (n)	Live birth (n)	Live birth rate (%)
Poor ovarian reserve	268	145	54.1
Poor oocyte/embryo quality	46	16	34.8
Oocyte maturation defect	6	4	66.7
Fertilization defect	9	4	44.4
Poor-quality cleavage-stage embryo*	26	8	30.8
Blastocyst formation defect	5	0	0.0
Genetic factor	63	29	46.0

* Patients exhibited zygotic cleavage failure, early embryonic arrest, or severe embryo fragmentation.

When recipients were grouped by age, LBR showed a gradual decreasing trend with increasing age (*P* for linear trend = 0.007) (see Supplementary Figure 1). The LBR for recipients aged 45 years and above was 42.5%. When grouped by cryopreservation duration, no significant differences were observed in survival rate, fertilization rate, cleavage rate, or day−3 usable embryo rate among groups (see Supplementary Table 1). Similarly, LBR did not differ significantly across groups (*P* for linear trend = 0.600) (see Supplementary Figure 2 A). RCS analysis confirmed no nonlinear association between oocyte cryopreservation duration and live birth (*P* for nonlinear trend = 0.101) (see Supplementary Figure 2B).

## Influencing factors of cumulative live birth

Among 369 patients who underwent the first embryo transfer, 80 received a second transfer and 13 received a third transfer after previous failures. The cumulative live birth rate (CLBR) was 58.5% (216/369) (see Supplementary Figure 3).

The analysis of influencing factors for cumulative live birth (as shown in Table 4)

**Table 4:** Logistic regression analysis of cumulative live birth.

	Estimate	OR (95%CI)	*P*
Recipient age	−0.072	0.93(0.87,1.00)	**0.041**
Recipient BMI	0.038	1.04(0.92,1.18)	0.557
Infertility years	0.013	1.01(0.97,1.06)	0.582
Infertility type	0.288	1.33(0.77,2.32)	0.302
Endometriosis	0.186	1.20(0.62,2.37)	0.585
Male age	0.019	1.02(0.96,1.08)	0.543
Severe oligoasthenospermia	−0.054	0.95(0.44,2.05)	0.890
Indication for donor oocyte use			
Poor ovarian reserve	Ref		
Poor oocyte/embryo quality	−0.845	0.43(0.22,0.82)	**0.011**
Genetic factor	−0.362	0.70(0.37,1.33)	0.271
Duration of oocyte cryopreservation	0.004	1.00(0.99,1.02)	0.557
Number of thawed oocytes	−0.104	0.90(0.74,1.10)	0.305
Number of day−3 usable embryos	0.388	1.47(1.26,1.73)	**0.000**

showed that advanced recipient age reduced CLBR (OR: 0.93; 95%CI: 0.87–1.00; *P* = 0.041). A history of poor oocyte/embryo quality also decreased CLBR (OR: 0.43; 95%CI: 0.22–0.82; *P* = 0.011). In addition, an increased number of day−3 usable embryos significantly improved CLBR (OR: 1.47; 95%CI: 1.26–1.73; *P* = 0.000).

Analysis of remaining embryos after the first live birth showed that increasing the number of thawed oocytes did not significantly improve LBR of the first embryo transfer or CLBR, but markedly increased the number of surplus embryos (*P* for linear trend = 0.000). When the number of thawed oocytes reached 6, the average number of remaining embryos after the first live birth was 1.50 (see Supplementary Figure 4).

## Discussion

This study demonstrated that advanced recipient age was associated with a reduced LBR in ART using vitrified donated oocytes, which is consistent with previous studies [10], [11]. Although oocyte donation provides young and high-quality oocytes, advanced maternal age may impair endometrial receptivity, leading to lower implantation and live birth rates [17]. A notable finding is that the decline in LBR with age was gradual, and recipients aged 45 years and older still achieved a clinically meaningful LBR, suggesting that oocyte donation remains a feasible and effective option for older women.

Recipients who received donated oocytes due to a history of poor oocyte/embryo quality had a significantly lower live birth rate (LBR) than those who received donated oocytes for other indications. As shown in Table 3, further subgroup analysis revealed that the significantly reduced LBR in this group was mainly driven by patients with a history of poor embryo quality, specifically those with poor-quality cleavage-stage embryos and blastocyst formation defect, who had extremely low live birth rates. Since oocyte donors were strictly selected and the quality of donated oocytes can be assumed to be consistent, the lower LBR in this subgroup was likely attributed to the effect of sperm quality on embryonic development [18]. Given that the ICSI technology allowed technicians to select sperm, it had largely reduced the impact of the routine concentration and morphology of sperm [19], [20], [21] on the pregnancy outcome. This study had not yet found that severe oligoasthenospermia affected the LBR. This implied that intrinsic sperm quality factors, such as sperm DNA integrity and epigenetics, may play a role [21], [22]. Therefore, this subgroup of patients with a history of poor embryo quality may benefit from interventions aimed at improving sperm quality, such as antioxidant therapy, or advanced sperm selection techniques to identify sperm with better developmental potential.

This study found that a higher number of day−3 usable embryos was associated with an increased CLBR, because more usable embryos provide more opportunities for subsequent embryo transfers. The relatively modest increase in CLBR in this study may be explained by the low rate of second embryo transfers (only 42.7% of patients with failed first transfer underwent a second transfer).

The number of thawed oocytes was not associated with LBR or CLBR, possibly because 90% of recipients received no more than 6 oocytes, limiting the analytical range. However, increasing the number of thawed oocytes significantly increased the number of surplus embryos after the first live birth. Given the scarcity of donated oocytes in China, a batch allocation strategy is advisable when six or more oocytes are available, thereby expanding access to donated oocytes for a greater number of patients.

Recipient BMI was not associated with LBR in this study, which may be due to the fact that 89.7% of recipients had a normal BMI, resulting in limited statistical power to detect potential effects.

Oocyte cryopreservation duration, up to 87 months, showed no adverse impact on laboratory or clinical outcomes, supporting the long-term safety and stability of oocyte vitrification [8], [9], [23]. Physicians and patients can be reassured that prolonged storage does not compromise oocyte developmental competence or pregnancy outcomes.

This study is a large-scale real-world study focusing on the Chinese population undergoing ART with vitrified donated oocytes under national regulations. The results of this study can provide important reference for the formulation of guidelines for oocyte donation in the local area. It has several limitations. First, due to local ethical and legal requirements mandating the anonymization of oocyte donor information, we were unable to adjust for donor-specific characteristics. However, only individuals meeting stringent donor criteria were eligible to become oocyte donors, and donors inherently represent a prognostically optimal population, which substantially reduces bias introduced by donor heterogeneity. Second, only a small number of recipients underwent sperm DNA integrity testing, so we could only speculate rather than confirm the role of sperm quality in the lower LBR among patients with poor embryo history. Finally, this was a single-center study, which may limit the generalizability of the results. Nevertheless, the standardized screening criteria and treatment protocols ensure the reliability and accuracy of the findings.

## Conclusions

This study comprehensively analyzed clinical outcomes and influencing factors of vitrified donated oocyte ART in Chinese patients. Advanced recipient age was associated with a gradual reduction in LBR. A history of poor oocyte/embryo quality was an independent risk factor for lower LBR. An increased number of day−3 usable embryos significantly improved CLBR. Oocyte vitrification is safe and not adversely affected by long-term storage. To alleviate the shortage of donated oocytes, a batch allocation strategy when six or more oocytes are available is recommended to improve utilization efficiency and expand access for more infertile families.

## CRediT authorship contribution statement

**Yangfeng Yu:** Writing – review & editing, Investigation. **Ge Lin:** Writing – review & editing, Supervision, Resources, Funding acquisition, Conceptualization. **Fei Gong:** Writing – review & editing, Supervision, Resources, Project administration, Conceptualization. **Qiuyun Yan:** Writing – review & editing, Investigation. **Juan Song:** Writing – review & editing, Investigation. **Xiaojuan Wang:** Writing – original draft, Project administration, Methodology, Formal analysis, Conceptualization. **Shuoping Zhang:** Writing – review & editing, Resources, Project administration, Investigation, Funding acquisition, Data curation, Conceptualization.

## Ethics approval and consent to participate

This study was approved by the Ethics Committee of the Reproductive and Genetic Hospital of CITIC-Xiangya, Changsha, People’s Republic of China (approval number, LL-SC−2025–013), and followed the Strengthening the Reporting of Observational Studies in Epidemiology reporting guideline. All donors provided written informed consent for the cryopreservation of a certain number of oocytes after oocyte retrieval. Subsequently, after they had used their own oocytes to generate multiple high-quality blastocysts, achieved a successful pregnancy, or delivered a healthy baby, they were advised to sign an informed consent form for donating their surplus frozen oocytes.

## Funding

The study was supported by Hunan Provincial Grant for Innovative Province Construction (2019SK4012), 10.13039/501100001809National Natural Science Foundation of China (22374146) and National Natural Science Foundation of China (62373076).

## Declaration of Competing Interest

The author(s) declare(s) that they have no competing interests.

## Appendix A Supplementary material

Supplementary material

Figure 1 Live birth rates by decile groups of recipient age

Figure 2 The relationship between oocyte cryopreservation duration and live birth. **2 A** Live birth rates by decile groups of oocyte cryopreservation duration (Test for linear association). **2B** The association between oocyte cryopreservation duration and live birth using restricted cubic spline functions (Test for nonlinear association). Odds ratios (ORs) were calculated using the median cryopreservation duration as the reference. The red curves represent ORs with 95% confidence intervals (CIs). The blue histogram showing recipient frequency across 10 equal-interval groups of oocyte cryopreservation duration

Figure 3 Treatment trajectories and outcomes for all transfer cycles

Figure 4 Live birth rates of the first embryo transfer, cumulative live birth rates, numbers of day 3 usable embryos and remaining embryos after first live birth by groups of thawed oocytes numbers

## References

[1] Qiao J., Wang Y., Li X., Jiang F., Hesketh T. (2021). A Lancet Commission on 70 years of women's reproductive, maternal, newborn, child, and adolescent health in China. Lancet.

[2] Gracia C., Anderson J., Flyckt R., Hill M., Jain T., Sakkas D. (2024). Gamete and embryo donation guidance. Fertil Steril.

[3] Yun S., Guo-Ning H., Hai-Xiang S., Li-Qing F., Yun F., Huan S. (2018). Egg-sharing donation program in China: an ethics committee opinion. J Reprod Med.

[4] Dyer S., Chambers G.M., Jwa S.C., Baker V.L., Banker M., de Mouzon J. (2025). International Committee for Monitoring Assisted Reproductive Technologies world report: assisted reproductive technology, 2019. Fertil Steril.

[5] Gliozheni O., Hambartsoumian E., Strohmer H., Petrovskaya E., Tishkevich O., De Neubourg D. (2023). ART in Europe, 2019: results generated from European registries by ESHRE. Hum Reprod.

[6] Cobo A., Meseguer M., Remohi J., Pellicer A. (2010). Use of cryo-banked oocytes in an ovum donation programme: a prospective, randomized, controlled, clinical trial. Hum Reprod.

[7] Glujovsky D., Riestra B., Sueldo C., Fiszbajn G., Repping S., Nodar F. (2014). Vitrification versus slow freezing for women undergoing oocyte cryopreservation. Cochrane Database Syst Rev.

[8] Huo Y., Yuan P., Qin Q., Yan Z., Yan L., Liu P. (2021). Effects of vitrification and cryostorage duration on single-cell RNA-Seq profiling of vitrified-thawed human metaphase II oocytes. Front Med.

[9] Torra-Massana M., Miguel-Escalada I., Vassena R., Rodríguez A. (2023). Long-term storage of vitrified oocytes does not affect pregnancy and live birth rates: analysis of 5362 oocyte donation cycles. Reprod Biomed Online.

[10] Chen T., Kuo P., Yu T., Wu M. (2024). IVF and obstetric outcomes among women of advanced maternal age (≥45 years) using donor eggs. Reprod Biomed Online.

[11] Yeh J.S., Steward R.G., Dude A.M., Shah A.A., Goldfarb J.M., Muasher S.J. (2014). Pregnancy outcomes decline in recipients over age 44: an analysis of 27,959 fresh donor oocyte in vitro fertilization cycles from the Society for Assisted Reproductive Technology. Fertil Steril.

[12] Cardozo E.R., Karmon A.E., Gold J., Petrozza J.C., Styer A.K. (2015). Reproductive outcomes in oocyte donation cycles are associated with donor BMI. Hum Reprod.

[13] Fabozzi G., Cimadomo D., Maggiulli R., Vaiarelli A., Badajoz V., Aura M. (2024). Association between oocyte donors’ or recipients’ body mass index and clinical outcomes after first single blastocyst transfers—the uterus is the most affected. Fertil Steril.

[14] Fitzgerald O., Newman J., Rombauts L., Polyakov A., Chambers G.M. (2024). Development of an IVF prediction model for donor oocytes: a retrospective analysis of 10 877 embryo transfers. Hum Reprod.

[15] Wang X., Zhang S., Gu Y., Ma S., Peng Y., Gong F. (2023). The impact of blastocyst freezing and biopsy on the association of blastocyst morphological parameters with live birth and singleton birthweight. Fertil Steril.

[16] Gardner D.K., Schoolcraft W.B. (1999). Vitr Cult Human blastocyst Towards Reprod Certain Infertil & Genet Beyond.

[17] Wang Y., Zhou P., Shan H., Liu X., Cheng M., Ye Z. (2025). Endometrial aging is accompanied by H3K27ac and PGR loss. Nat Aging.

[18] Cozzolino M., Hervás I., Rivera-Egea R., Pellicer A., Garrido N. (2021). Do donor spermatozoa improve reproductive outcomes after oocyte donation failure? A retrospective analysis of cumulative live birth rates per donor oocyte consumed. Reprod Biomed Online.

[19] Cozzolino M., Pellegrini L., Ottolini C.S., Capalbo A., Galliano D., Pellicer A. (2025). The clinical impact of oligozoospermia in oocyte donation ICSI cycles using preimplantation genetic test for aneuploidy. Hum Reprod.

[20] Loutradi K.E., Tarlatzis B.C., Goulis D.G., Zepiridis L., Pagou T., Chatziioannou E. (2006). The effects of sperm quality on embryo development after intracytoplasmic sperm injection. J Assist Reprod Genet.

[21] Zhou W.J., Huang C., Jiang S.H., Ji X.R., Gong F., Fan L.Q. (2021). Influence of sperm morphology on pregnancy outcome and offspring in in vitro fertilization and intracytoplasmic sperm injection: a matched case-control study. Asian J Androl.

[22] Li F., Duan X., Li M., Ma X. (2024). Sperm DNA fragmentation index affect pregnancy outcomes and offspring safety in assisted reproductive technology. Sci Rep.

[23] Cobo A., Garrido N., Pellicer A., Remohí J. (2015). Six years' experience in ovum donation using vitrified oocytes: report of cumulative outcomes, impact of storage time, and development of a predictive model for oocyte survival rate. Fertil Steril.

