# Supplementary material for: Influencing factors of live birth in vitrified oocyte donation cycles: A retrospective cohort study
Source: Eur J Obstet Gynecol Reprod Biol X. 2026 Jun 27;31:100473. doi: 10.1016/j.eurox.2026.100473 (PMC13342946; doi:10.1016/j.eurox.2026.100473)
Supplement: Supplementary file 1 — Supplementary material [file mmc1.docx]

Supplementary Table 1 Laboratory outcomes of donated oocytes by groups based on deciles of oocyte cryopreservation duration

| Group | Recipients (n) | Thawed oocytes (n) | Survival oocytes (n) | Fertilized oocytes (n) | Cleavage embryos (n) | Day-3 usable embryos (n) | Survival rate (%) | Fertilization rate (%) | Cleavage rate (%) | Day-3 usable embryo formation rate (%) |
| --- | --- | --- | --- | --- | --- | --- | --- | --- | --- | --- |
| G1 | 38 | 239 | 234 | 171 | 168 | 138 | 97.9 | 73.1 | 98.2 | 80.7 |
| G2 | 41 | 245 | 231 | 185 | 180 | 155 | 94.3 | 80.1 | 97.3 | 83.8 |
| G3 | 37 | 210 | 195 | 147 | 145 | 129 | 92.9 | 75.4 | 98.6 | 87.8 |
| G4 | 39 | 206 | 200 | 165 | 165 | 143 | 97.1 | 82.5 | 100 | 86.7 |
| G5 | 44 | 235 | 225 | 175 | 169 | 155 | 95.7 | 77.8 | 96.6 | 88.6 |
| G6 | 31 | 173 | 163 | 127 | 123 | 108 | 94.2 | 77.9 | 96.9 | 85.0 |
| G7 | 42 | 239 | 231 | 184 | 182 | 161 | 96.7 | 79.7 | 98.9 | 87.5 |
| G8 | 32 | 188 | 181 | 152 | 147 | 124 | 96.3 | 84.0 | 96.7 | 81.6 |
| G9 | 36 | 203 | 197 | 155 | 151 | 126 | 97.0 | 78.7 | 97.4 | 81.3 |
| G10 | 37 | 202 | 186 | 146 | 144 | 124 | 92.1 | 78.5 | 98.6 | 84.9 |
| *P* for linear trend test | | |  |  |  |  | 0.620 | 0.217 | 0.711 | 0.835 |
