# Supplementary figures and images for: Influencing factors of live birth in vitrified oocyte donation cycles: A retrospective cohort study
Source: Eur J Obstet Gynecol Reprod Biol X. 2026 Jun 27;31:100473. doi: 10.1016/j.eurox.2026.100473 (PMC13342946; doi:10.1016/j.eurox.2026.100473)

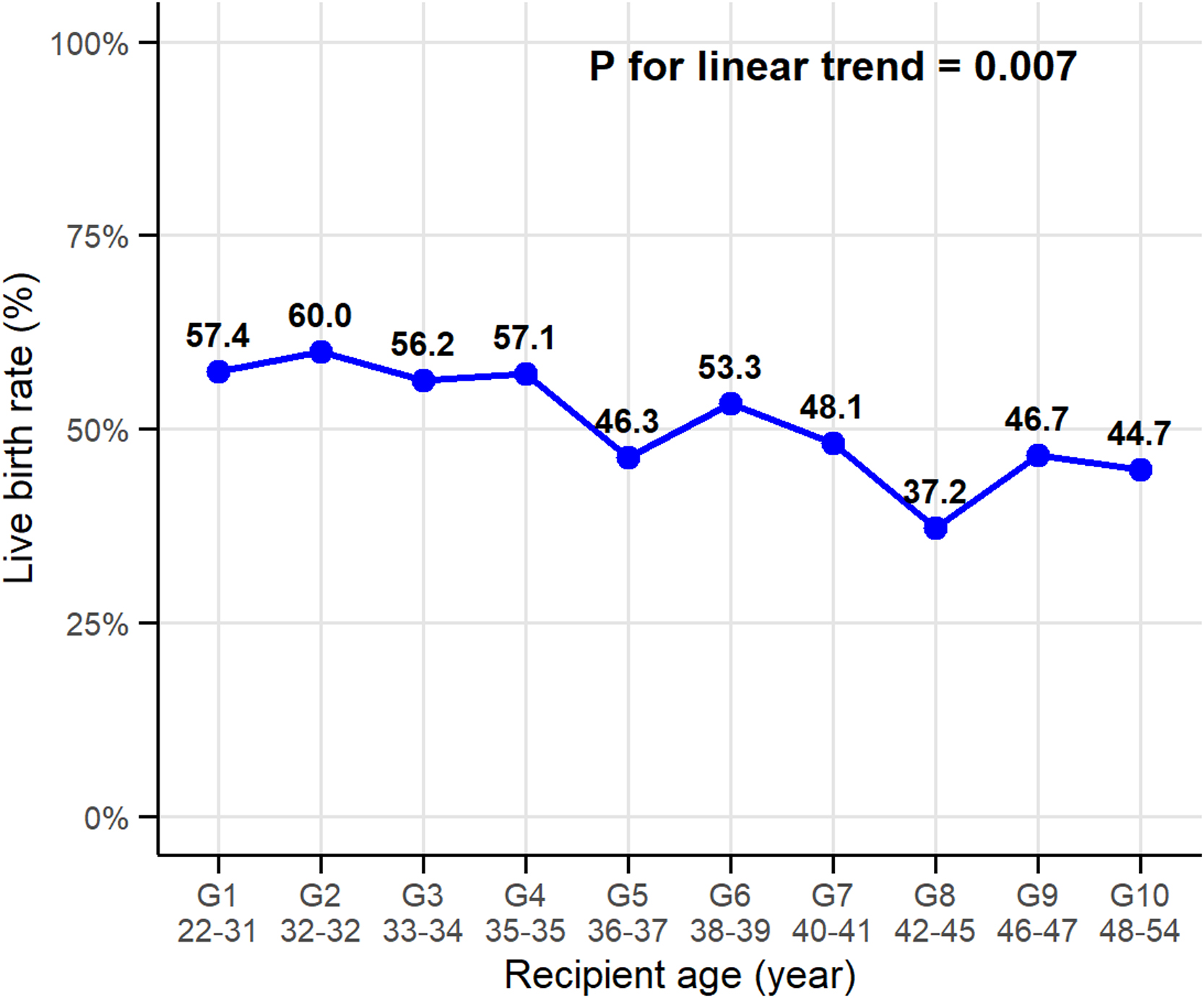

Supplement: Supplementary file 2 — Figure 1 Live birth rates by decile groups of recipient age [file mmc2.jpg]

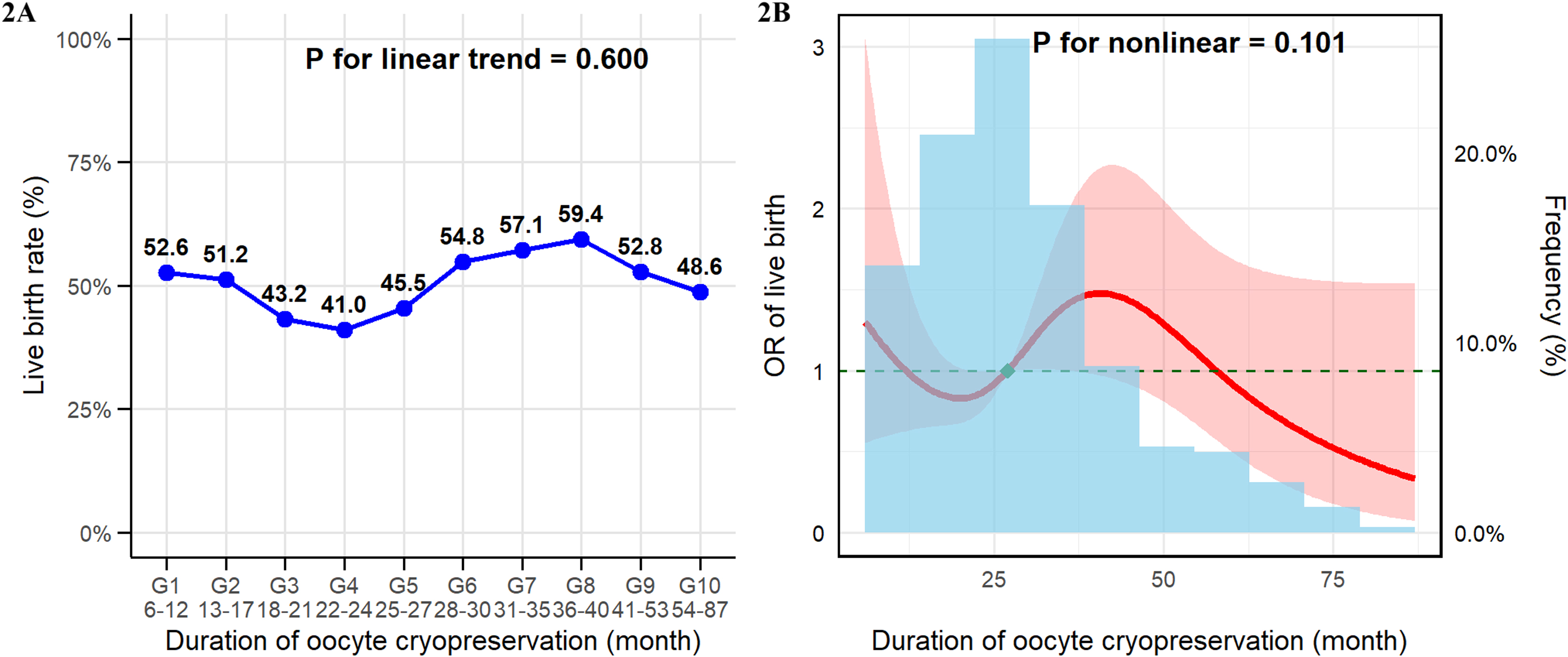

Supplement: Supplementary file 3 — Figure 2 The relationship between oocyte cryopreservation duration and live birth. 2 A Live birth rates by decile groups of oocyte cryopreservation duration (Test for linear association). 2B The association between oocyte cryopreservation duration and live birth using restricted cubic spline functions (Test for nonlinear association). Odds ratios (ORs) were calculated using the median cryopreservation duration as the reference. The red curves represent ORs with 95% confidence intervals (CIs). The blue histogram showing recipient frequency across 10 equal-interval groups of oocyte cryopreservation duration [file mmc3.jpg]

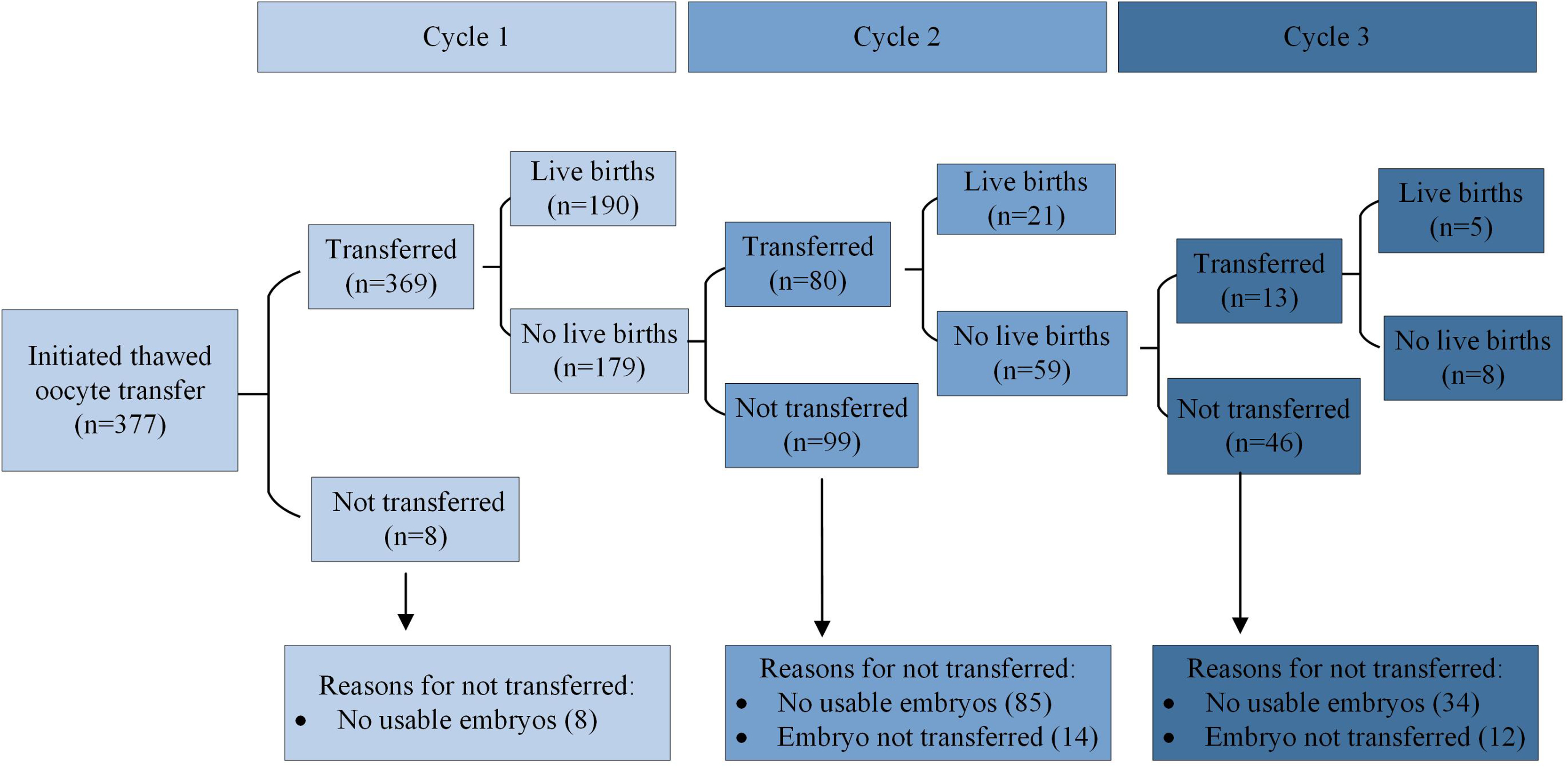

Supplement: Supplementary file 4 — Figure 3 Treatment trajectories and outcomes for all transfer cycles [file mmc4.jpg]

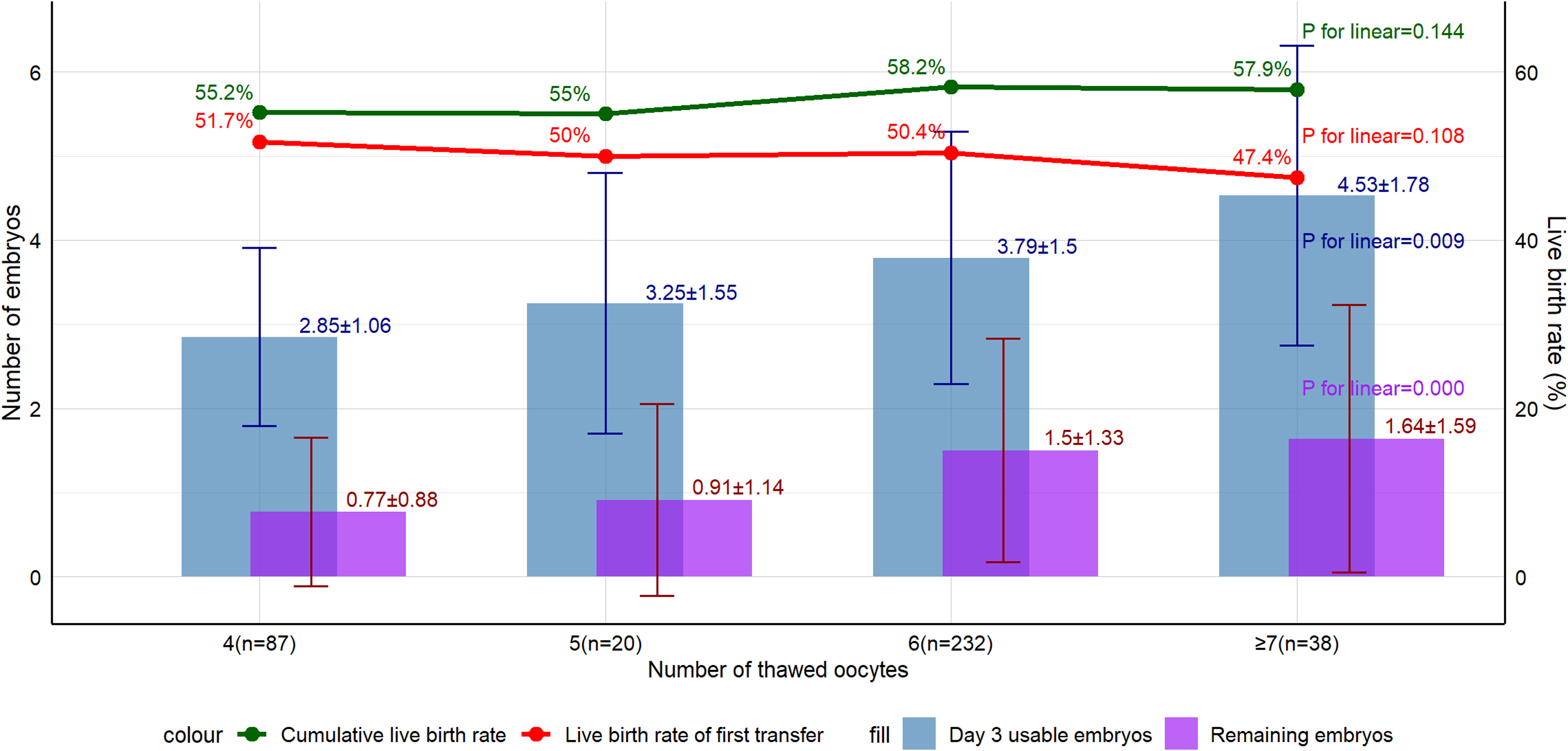

Supplement: Supplementary file 5 — Figure 4 Live birth rates of the first embryo transfer, cumulative live birth rates, numbers of day 3 usable embryos and remaining embryos after first live birth by groups of thawed oocytes numbers [file mmc5.jpg]
